# High Expression of Microtubule-associated Protein TBCB Predicts Adverse Outcome and Immunosuppression in Acute Myeloid Leukemia

**DOI:** 10.7150/jca.84215

**Published:** 2023-06-12

**Authors:** Bichen Wang, Wenjun Wang, Qiaoli Li, Tengxiao Guo, Shuang Yang, Jun Shi, Weiping Yuan, Yajing Chu

**Affiliations:** 1 State Key Laboratory of Experimental Hematology, National Clinical Research Center for Blood Diseases, Haihe Laboratory of Cell Ecosystem, Institute of Hematology & Blood Diseases Hospital, Chinese Academy of Medical Sciences & Peking Union Medical College, Tianjin, 300020, China.; 2 Tianjin Institutes of Health Science, Tianjin 301600, China.; 3 Regenerative Medicine Clinic, Institute of Hematology & Blood Diseases Hospital, Chinese Academy of Medical Sciences & Peking Union Medical College, Tianjin, 300020, China.

**Keywords:** TBCB, prognosis, acute myeloid leukemia, bioinformatics, immune evasion, drug sensitivity

## Abstract

Acute myeloid leukemia (AML) is a devastating blood cancer with high heterogeneity and ill-fated outcome. Despite numerous advances in AML treatment, the prognosis remains poor for a significant proportion of patients. Consequently, it is necessary to accurately and comprehensively identify biomarkers as soon as possible to enhance the efficacy of diagnosis, prognosis and treatment of AML. In this study, we aimed to identify prognostic markers of AML by analyzing the cohorts from TCGA-LAML database and GEO microarray datasets. Interestingly, the transcriptional level of microtubule-associated protein TBCB in AML patients was noticeably increased when compared with normal individuals, and this was verified in two independent cohorts (GSE9476 and GSE13159) and with our AML patients. Furthermore, univariate and multivariate regression analysis revealed that high *TBCB* expression was an independent poor prognostic factor for AML. GO and GSEA enrichment analysis hinted that immune-related signaling pathways were enriched in up-regulated DEGs between two populations separated by the median expression level of *TBCB*. By constructing a protein-protein interaction network, we obtained six hub genes, all of which are immune-related molecules, and their expression levels were positively linked to that of *TBCB*. In addition, the high expression of three hub genes was significantly associated with a poor prognosis in AML. Moreover, we found that the tumor microenvironment in AML with high *TBCB* expression tended to be infiltrated by NK cells, especially CD56^bright^ NK cells. The transcriptional levels of NK cell inhibitory receptors and their ligands were positively related to that of *TBCB*, and their high expression levels also predicted poor prognosis in AML. Notably, we found that the down-regulation of TBCB suppressed cell proliferation in AML cell lines by enhancing the apoptosis and cell cycle arrest. Finally, drug sensitivity prediction illustrated that cells with high *TBCB* expression were more responsive to ATRA and midostaurin but resistant to cytarabine, dasatinib, and imatinib. In conclusion, our findings shed light on the feasibility of *TBCB* as a potential predictor of poor outcome and to be an alternative target of treatment in AML.

## Introduction

As a quickly and dramatically progressing heterogeneous blood cancer, acute myeloid leukemia (AML) derived from blockage in differentiation and clonal expansion of myeloid blasts 1, 2. AML patients are often accompanied by weakness, hemorrhage, infection and other complications. Traditional anthracycline and cytarabine chemotherapy (7+3 regimen) has limited efficacy 3, while leukemia relapse is common after allogeneic hematopoietic stem cell transplantation (HSCT) leads to unfavorable prognosis 4. Lately, the development of several new therapies based on personalized biomarkers in AML has played a positive role in strengthening the efficacy of systemic therapy and ameliorating the survival of patients to a certain extent 5, 6. Nevertheless, the intrinsic and acquired drug-resistance are still observed in AML patients 7. Large-scale genomic and transcriptomic analyses have furnished profound perspective for the molecular landscape of AML and identified potential targets for precision medicine. However, the identification of reliable and robust biomarkers remains a challenge, given the complexity and heterogeneity of AML.

Tumors proliferate rapidly due to their frequent mitoses and shortened cell cycle 8. In all eukaryotic cells, microtubules are crucial components of the cytoskeleton, that can be assembled with other proteins into spindles, centriole and neural tube 9, 10. Because of the central role in cell growth, maintenance of morphology, cell signal transduction, microtubules have also become a key target for tumor drug therapy 11. Microtubule targeted agents (MTAs) exert anti-tumor effects by altering microtubule dynamics and thus affecting the mitotic process of tumor cells 12, 13. MTAs have been used to treat various tumors 14 including leukemia 15, lymphoma 16 and multiple myeloma (MM) 17. Tubulin binding cofactors (TBCs) play an important role in regulating microtubule folding and α/β-tubulin dimer formation 18. High TBCA expression impacts the cell proliferation, invasion and metastasis on renal clear cell carcinoma 19. MiR-1251-5p promotes carcinogenesis in ovarian cancer cells by directly targeting TBCC 20. TBCE, preferentially expressed in CML CD34^+^ cells, is a potential target antigen for immunotherapy of CML 21. Nevertheless, the latent role of TBCs in AML and if they can be used as therapeutic and prognostic biomarkers for AML are still unclear.

As a microtubule folding cofactor, TBCB is mainly localized in the cytoplasm and centrosomes 22. The human TBCB consists of 244 amino acids and contains two spherical functional domains, namely the ubiquitin-like domain in the N-terminal and the CAP-Gly domain in the C-terminal 23. CAP-Gly domain is the recognition region of EEY/F-COO-peptide, although the specific function is not clear 24. Interestingly, previous studies have shown that this CAP-Gly domain could be self-inhibited by interacting with the last three residues of its C-terminus 22. In addition, the last three residues of TBCB are also required for the recognition and interaction with TBCE, and tubulin heterodimer dissociation 22. In this context, we focused on the expression levels of microtubule-associated protein *TBCB* in AML patients, which were noticeably increased when compared with those of normal individuals, and found that elevated level of *TBCB* was an unaided poor prognostic factor. Moreover, we interrogated the relevance between *TBCB* expression and immune infiltration, expression levels of immune-related molecules, and drug sensitivity in AML.

## Material and methods

### Data source and processing

In this study, six independent cohorts for mRNA expression were used, namely the TCGA-LAML (AML, n = 173) 25 and GTEx datasets (Normal, n=70) 26, the GSE9476 microarray dataset (AML, n = 25; Normal, n = 10) 27, the GSE13159 microarray dataset (AML, n = 503; Normal, n = 72) 28, 29, the GSE37642-GPL570 microarray dataset (AML, n = 136) 30, the GSE71014 (AML, n = 104) 31 and the GSE12417-GPL570 microarray dataset (AML, n = 78) 32. The TPM values of RNA-Seq were log_2_ transformed for intrasample comparison. The data and information of the datasets (TCGA-LAML and GTEx) are available searched on the UCSC XENA 33. In addition, the GEO database was adapted into getting above microarray data.

### Acquisition of clinical samples in our own cohort

Bone marrow mononuclear cells from AML patients and health subjects were extracted by Ficoll-Paque (10771, Sigma-Aldrich, USA) density gradient centrifugation. The inclusion criteria in this study were: (1) patients with confirmed diagnosis of AML according to WHO-2022 34, (2) newly diagnosed patients, (3) ECOG performance status ≤ 2, (4) age ≥ 18 years. Exclusion criteria were: (1) acute promyelocytic leukemia (AML M3), (2) patients with Down syndrome, (3) patients with known central nervous system manifestation of AML, (4) evidence or history of severe non-leukemia related bleeding diathesis or coagulopathy, (5) isolated extramedullary manifestation of AML, (6) uncontrolled or significant disease of vital organs, including heart, lung, liver, kidney and so on. The age of the study subjects ranged from 31 to 71 years. All patients' information was queried in Table S1.

### Cell culture

The human AML cell lines (THP1, NOMO1, Kasumi-1 and SKNO-1) were obtained from State Key Laboratory of Experimental Hematology. THP1, NOMO1 and Kasumi-1 cells were cultured at 1-2 × 10^6^ cells/mL in RPMI-1640 (C11875500BT, GIBCO, USA) containing 10% fetal bovine serum (FBS, C04001, Vivacell, China), 1% penicillin/streptomycin (SV30010, HyClone, USA). In addition to the above components, cell culture medium for SKNO-1 was supplemented with 10 ng/ml GM-CSF (300-03, PeproTech, USA). Cells were maintained at 37°C in a humidified incubator (ThermoFisher, USA).

### Small-interfering RNAs (siRNA) transfection

The siRNA oligonucleotides targeting TBCB (siTBCB) or negative control (NC-siRNA) were synthesized by Suzhou GenePharma Co.,Ltd (Suzhou, China) and transfected into AML cell lines by Lipofectamine™ 3000 transfection kit (L3000015, Invitrogen, USA). Each well of a 6-well plate contained 1 × 10^6^ cells, 5 μL siRNA, 5 μL Lipofectamine 3000, 500 μL cell culture medium and 500 μL Opti-MEM (31985070, Invitrogen, USA). Transfected cells were harvested for analysis 48 hours after siRNA transfection, including validation of TBCB expression level by RT-qPCR and Western blot, and detection of apoptosis and cell cycle by flow cytometry. The siRNA sequences (5'→3') were as follows: TBCB-siRNA 1: forward GCAUCCACGUCAUUGACCATT, reverse UGGUCAAUGACGUGGAUGCTT; TBCB-siRNA 2: forward GGGAAACGCUACUUCGAAUTT, reverse AUUCGAAGUAGCGUUUCCCTT; and NC-siRNA: forward UUCUCCGAACGUGUCACGUTT, reverse ACGUGACACGUUCGGAGAATT.

### Verification of *TBCB* transcriptional level by RT-qPCR

TRIzol (15596018, Invitrogen, USA) and Phasemaker^TM^ Tubes (A33248, Invitrogen, USA) were used to extract total RNA 35. Reverse transcription kits (RR047A, TAKARA) were used to synthesize cDNA. Real-time quantitative PCR (RT-qPCR) was performed using FastStart Universal SYBR Green Master (4913914001, Roche, Switzerland) on a Real-time PCR instrument (Quanstudio 5, ThermoFisher, USA). ∆Ct values were calculated using 18S as control. Primer sequences (5'→3') were as follows: h-*TBCB*-forward, CTACTGGATTGGTGTCCGCTATG, h-*TBCB*-reverse, CACGACTGCTGGCTTGACAAAG; 18S-forward, AGTCCCTGCCCTTTGTACACA, 18S-reverse, CGATCCGAGGGCCTCACTA.

### Western blot

Western blot analysis was performed as previously described 36. The cells were washed with pre-chilled PBS and lysed with 1 × SDS-PAGE sample loading buffer (#E153-01, GenStar, China) at room temperature (RT) for 30 minutes, followed by boiling for 10 minutes at 100°C. The proteins were separated on 12.5% SDS-PAGE gels (PG113, Epizyme Biotech, China) and transferred to 0.2-μm polyvinylidene difluoride (PVDF) membranes (ISEQ00010, Millipore, Germany). The membranes were blocked with 3% nonfat dry milk at RT for one hour and incubated overnight at 4°C with primary antibodies at the appropriate dilutions. Subsequently, they were incubated with secondary antibodies for one hour at RT and detected by a Bio-Rad ChemiDoc (Bio-Rad, USA). The primary antibodies were used in this study as follow: anti-TBCB (A13248, Abclonal, China) and anti-β-Actin (3700S, Cell signaling technology, USA). All primary antibodies were used at a 1:1000 dilution, and the secondary antibodies (Jackson ImmunoResearch, USA) were used at a 1:10000 dilution.

### Cell counting Kit-8 (CCK-8) assay

The CCK-8 assay was performed to assess cell proliferation. Transfected cells were seeded into 96-well plates at 2 × 10^3^ cells/well. 10 μL of CCK-8 reagent (40203ES80, YEASEN, China) was added to the wells after 24 h, 48 h and 72 h, and incubated for 4 hours at 37°C. Absorbance at 450 nm was measured using a microplate spectrophotometer (Bio-Tek, USA).

### Cell apoptosis analysis

The Annexin V Apoptosis Detection Kit (88-8102-72, eBioscience, USA) was used to analyze cell apoptosis. Specifically, cells were washed once with PBS and once with 1 × Binding Buffer. Then, cells were resuspended in 1 × Binding Buffer at 2 × 10^5^/50 μL. 3 μL Annexin V and 3 μL 7-AAD were added to the cell suspension and incubated for 15 minutes at RT in the dark. After that, 200 μL of 1 × Binding Buffer was added to the cell suspension and cells were analyzed by a FACS Canto II Flow Cytometry (BD).

### Cell cycle analysis

Cell cycle progression was measured using APC-Ki67 (17-5698-82, Invitrogen, USA) and Hoechst 33342 (14533, Sigma-Aldrich, USA). BD IntraSure^TM^ kit (641776, BD, USA) was used to fix and permeabilize the cells. Brieflly, cells were washed with PBS and vigorously resuspended in Reagent A at 3 × 10^5^/100 μL. After incubation for 5 minutes at RT, 1 mL 1 × BD FACS lysing solution was added, vortexed vigorously and incubated 10 minutes at RT. Then, cells were centrifugated at 2000 rpm for 5 min and the pellets were resuspended in 50 μL Reagent B. After that, 3 μL Ki67 was added to the cell suspension and incubated for 30 minutes at RT in the dark. Finally, cells were labelled with 20 ng/μL Hoechst 33342 before analyzed by FACS Canto II Flow Cytometry (BD).

### Differentially expressed genes (DEGs) analysis

Patients were split into two groups gleaned from the median *TBCB* expression level. DESeq2 37 was used for identifying DEGs with default parameters and the following thresholds: *p*-adjust < 0.05, |log_2_FC| ≥ 0.59. The data was originated from TCGA-LAML.

### GO and KEGG enrichment analysis

ClusterProfiler (v4.4.4) and GOplot (v1.0.2) were used to carry out functional annotation analysis of DEGs 38, 39. Terms were interpreted as statistically significant if the Benjamini-Hochberg *p*-adjust < 0.05.

### Gene set enrichment analysis (GSEA)

ClusterProfiler (v4.4.4) was used to conduct GSEA by the C2 gene sets (c2.cp.all.v2022.1.Hs.symbols.gmt) from the MSigDB database 38, 40. Results with |NES| >1.5, FDR < 0.05 and *p*-adjust < 0.05 were defined as statistical significance.

### Establishment of protein interaction network

Raw data of protein-protein interactions (PPIs) network to assess the interrelationships of the up-regulated DEGs were downloaded from STRING 39. The interaction possesses a greater than 0.4 confidence score was defined as significant. Three algorithms (MCC, MNC and EPC) in Cytoscape (v3.9.1) 41 were applied to recognize and visualize the pivotal hub genes. Statistical comparisons were performed by Spearman correlation. Correlations between transcriptional level of *TBCB* and those of hub genes in AML subjects were visualized using ggplot2 (v3.3.6).

### Correlation with immune infiltrating cells and immune checkpoint molecules

Based on the ssGSEA algorithm provided in the GSVA (v1.46.0) 42, the immune cells (a total of twenty-four) from previous research 43 were used to estimate the immune infiltration. Then the relationship of *TBCB* mRNA level and that of immune checkpoint molecules were identified and visualized via ggplot2 (v3.3.6).

### Prediction of drug sensitivity

The pRRophetic 44 was used to calculate the median inhibition concentration (IC50) values for drug sensitivity prediction 5.

### Statistical analysis

The R-studio (v4.2.1) was used for most data processing and statistical analysis. The Kruskal-Wallis test (multiple groups) and Wilcoxon rank sum test (unpaired samples of two groups) were applied to define the differences in clinical characteristics. Unpaired t test was used to compare the means of the two groups for RT-qPCR and drug sensitivity analysis. One-way ANOVA was used to assess differences among multiple groups in biologically experiments. The overall survival (OS) was conducted by Kaplan-Meier, and univariate and multivariate Cox regression were used to determine the significance of prognostic factors (*p* < 0.05). Receiver operating characteristic (ROC) analysis was carried out on the data using the pROC package (v1.18.0) and the results were visualized using ggplot2 (v3.3.6). Hazard ratios (HR) and their 95% confidence intervals (CI) were computed for survival-related genes.

## Results

### High *TBCB* expression in AML is prevalent with adverse clinical features

**Figure 1:**
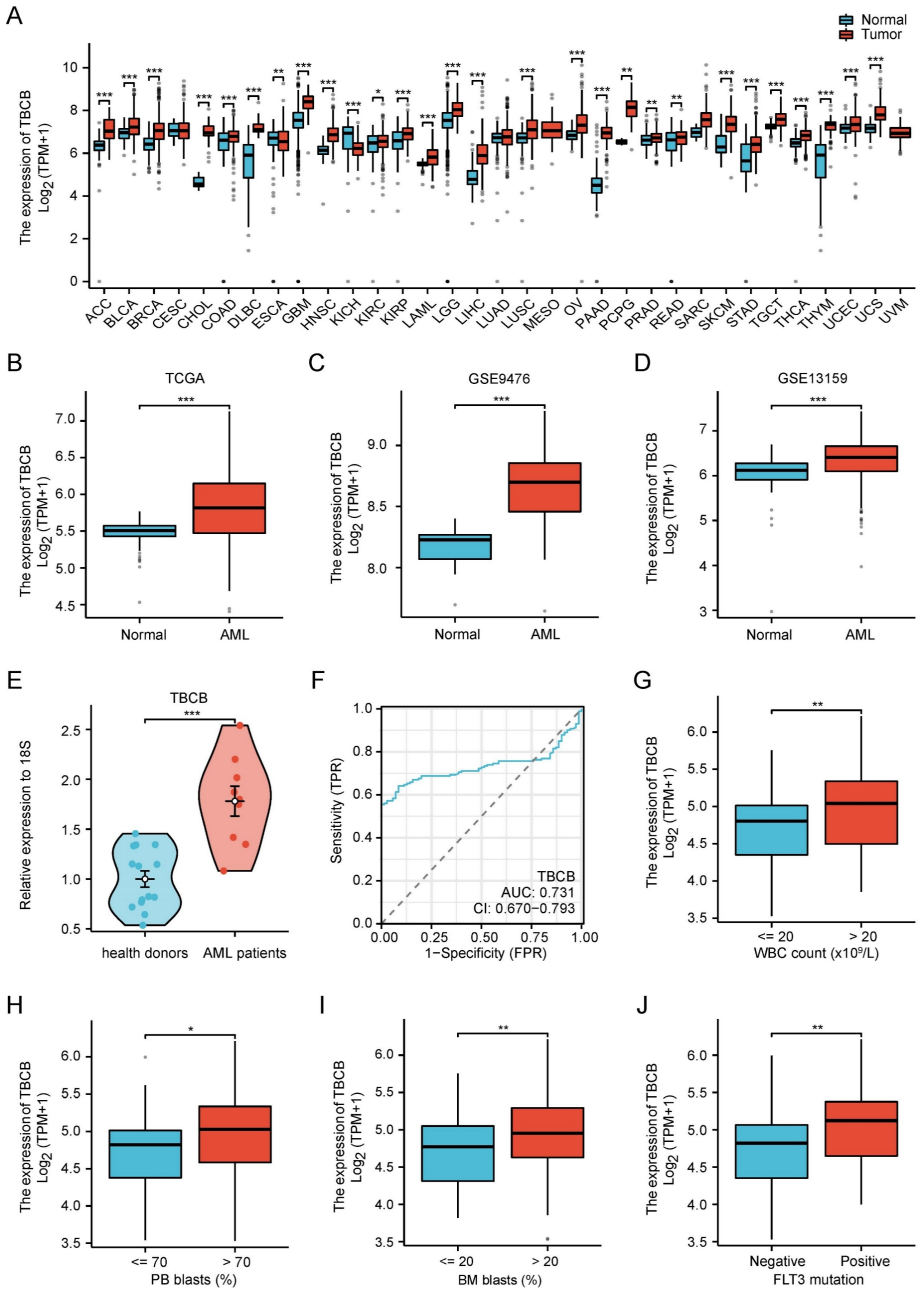
The clinical features of AML patients highly expressed *TBCB*. (A) Transcriptional levels of *TBCB* in thirty-three different cancers. Data was originated from TCGA and GTEx database. (B) The *TBCB* mRNA levels of AML patients in comparison with normal subjects. Data was originated from TCGA-LAML and GTEx database. (C-D) The differentially expression of *TBCB* between AML patients and normal subjects from GSE9476 (C) and GSE13159 (D). (E) RT-qPCR analysis for transcriptional levels of *TBCB* in BMMNC from AML patients (n = 9) relative to healthy donors (n = 14). Expression levels are normalized to 18S. (F) ROC analysis of *TBCB* in TCGA-LAML and GTEx datasets. (G-J) Significant clinical features were demonstrated, including count of white blood cells (G), proportions of PB blasts (H) and BM blasts (I), mutation rate of FLT3 (J). *p < 0.05; **p < 0.01; ***p < 0.001.

We firstly determined the expression profile of TBCs by analyzing the transcriptional levels in AML patients and normal subjects that were acquired from TCGA-LAML and GTEx datasets. Among five members of the TBC family (TBCA, TBCB, TBCC, TBCD, TBCE), only the transcriptional level of *TBCB* was increased in AML, while the others were decreased (Figure S1A). Higher *TBCB* expression was observed in most tumors using pan-cancer analysis (Figure 1A), including AML (*p* < 0.001, Figure 1B), verified using microarray datasets from GEO (GSE9476, *p* < 0.001, Figure 1C; GSE13159, *p* < 0.001, Figure 1D). To further validate the expression level of *TBCB* in AML, we examined the transcriptional levels of *TBCB* via RT-qPCR in bone marrow mononuclear cells from AML patients (n = 9) and healthy donors (n = 14). Consistent with the expression profile analysis with database, we noticed a substantially augmentation of transcriptional level of *TBCB* in AML patients (*p* < 0.001, Figure 1E). Additionally, the mRNA levels of *TBCB* were also evidently increased in human AML cell lines compared with cord blood CD34^+^ cells (control) (Figure S1B).

We then depicted the ROC curves to assess the ability to discriminate between AML patients and normal individuals based on *TBCB* expression. The calculated area under curve (AUC) value was 0.731 (Figure 1F), hinting that *TBCB* was a potential marker for distinguishing AML patients from normal samples. To determine if the transcriptional levels of *TBCB* are clinically relevant in AML, we analyzed AML samples with complete clinical information from TCGA-LAML. Surprisingly, the high expression level of *TBCB* was closely linked to increased white blood cell (WBC) counts (*p* < 0.01, Figure 1G), augmented proportions of blasts in peripheral blood (PB) (*p* < 0.05, Figure 1H) and bone marrow (BM) (*p* < 0.01, Figure 1I), and FLT3 positive mutation (*p* < 0.01, Figure 1J), while no association was observed in gender, age, cytogenetic risk or French-American-British (FAB) classifications (Table 1 and Figure S1C-F).

### High expression of *TBCB* is predictive for poor outcome in AML

**Figure 2:**
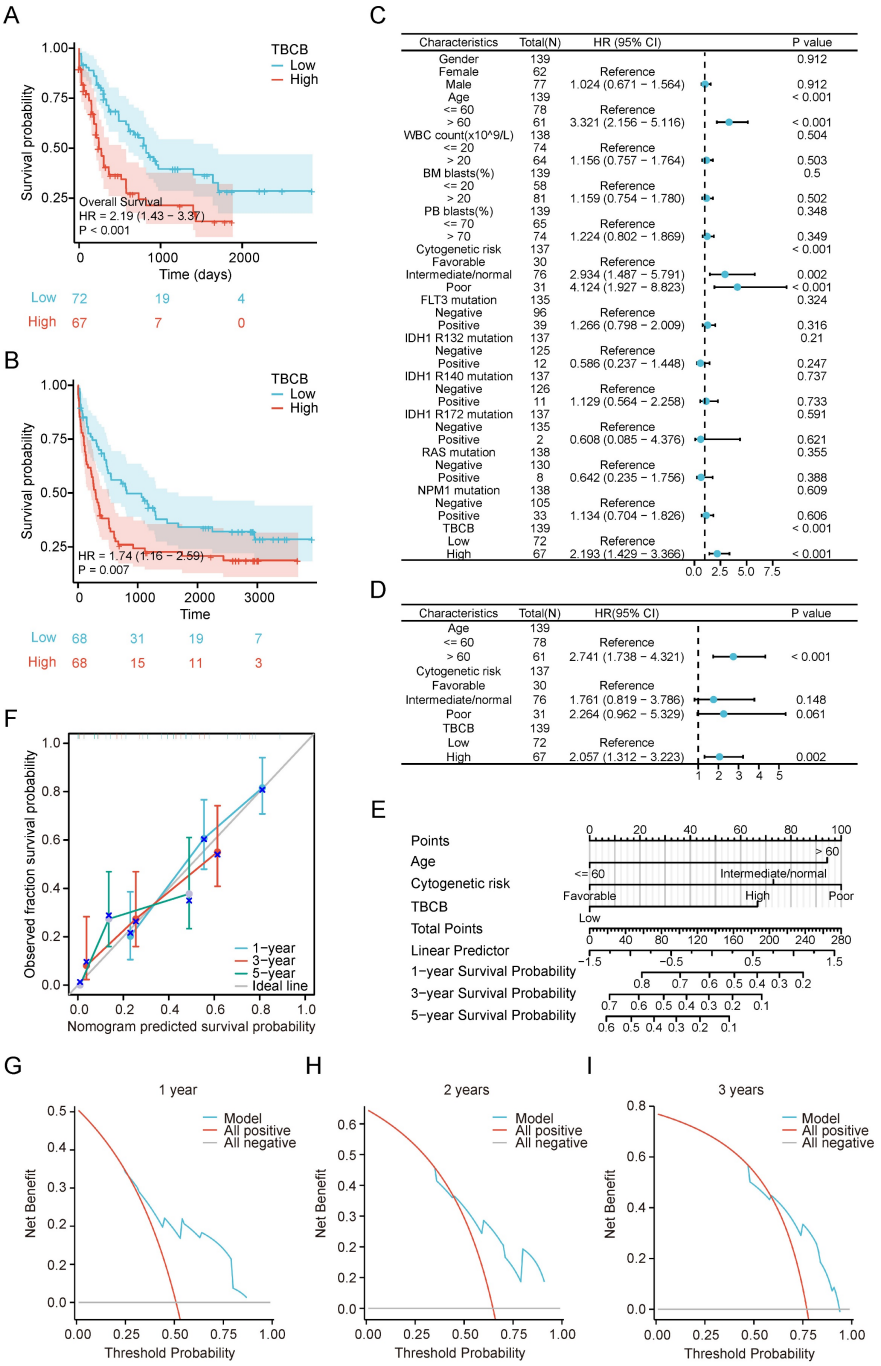
High *TBCB* expression correlated with unfavorable prognosis. (A) Kaplan-Meier survival curve of OS was delineated for AML patients grouped into high versus low expressed populations in line accordance with the median expression of *TBCB*. Data was originated from TCGA-LAML dataset. (B) Validation of OS for *TBCB* in the entirely independent cohort GSE37642 (n = 136). (C-D) Forest plots of OS for AML patients from univariate (C) and multivariable (D) analysis. (E) Nomogram based on integrating *TBCB* and other meaningful prognostic factors of AML. (F) Calibration of the nomogram. (G-I) The DCA curves of the nomogram at 1 year (G), 2 years (H), and 3 years (I).

Subsequently, to investigate the prognostic implication of TBC family in AML, we used the clinical information from TCGA-LAML to calculate the overall survival (OS) curve. The group with high *TBCB* (*p* < 0.001; Figure 2A) and* TBCC* (*p* = 0.001; Figure S1G) expression had worse survival than the low expression group, while there were no remarkably disparities in prognosis of patients grouped based on the expression levels of other members (Figure S1H-J). Consistent with above findings, an unfavorable prognosis in AML patients with high mRNA levels of *TBCB* was exhibited in the independent external validation cohort, GSE37642 (AML, n = 134; *p* = 0.007; Figure 2B). Although there was no significant difference between the two groups of high or low *TBCB* expression level in AML patients from other two GEO datasets (GSE12417 and GSE71014), there was a trend that *TBCB* high expression maybe indictive of poor prognosis of AML (Figure S1K-L). In addition to grouping by median expression level of *TBCB*, we also split by quartile groups, and found that the results were similar to the above findings (Figure S1M-P). The combined data that expression levels and correlation coefficient with survival of AML patients led us to postulate that *TBCB* may be a potential prognostic marker for AML.

Furthermore, we estimated whether the high *TBCB* expression is an independent unfavorable prognostic factor for AML by logistic regression analyses. Univariate Cox regression analysis illustrated that advanced age (*p* < 0.001), unfavorable cytogenetic risk (intermediate, *p* = 0.002; poor, *p* < 0.001) and higher *TBCB* expression (*p* < 0.001) were associated with adverse outcome (Figure 2C). Multivariate Cox regression analysis was accomplished through the obvious variables (*p* < 0.01) from univariate Cox regression analysis, and we found that older age (*p* < 0.001) and high *TBCB* expression (*p* = 0.002) were independent prognostic factors with clinical meaning for OS (Figure 2D and Table S2).

Then, a nomogram model was constructed with age, cytogenetic risk and *TBCB* expression, which were independent factors in univariable analysis. The built nomogram possessed good calibration and discrimination, and the Concordance index (CI) of OS prediction was 0.738 (*p* < 0.001; Figure 2E).

Furthermore, to describe the clinical net benefit, we plotted the calibration curves, that showed the short-term (1 or 3 years) calibration curve presented high concordance between the nomogram predicted survival probability and observed fraction survival probability (Figure 2F). To enhance the credibility of this prediction model for AML prognosis, we performed the decision curve analysis for the individualized prediction nomogram in the short term (1 to 3 years, Figure 2G-I). In summary, the constructed survival-predictive model revealed excellent accuracy for predicting OS in AML patients.

### GO and GSEA analysis for DEGs were enriched in immune-related signaling pathways

**Figure 3:**
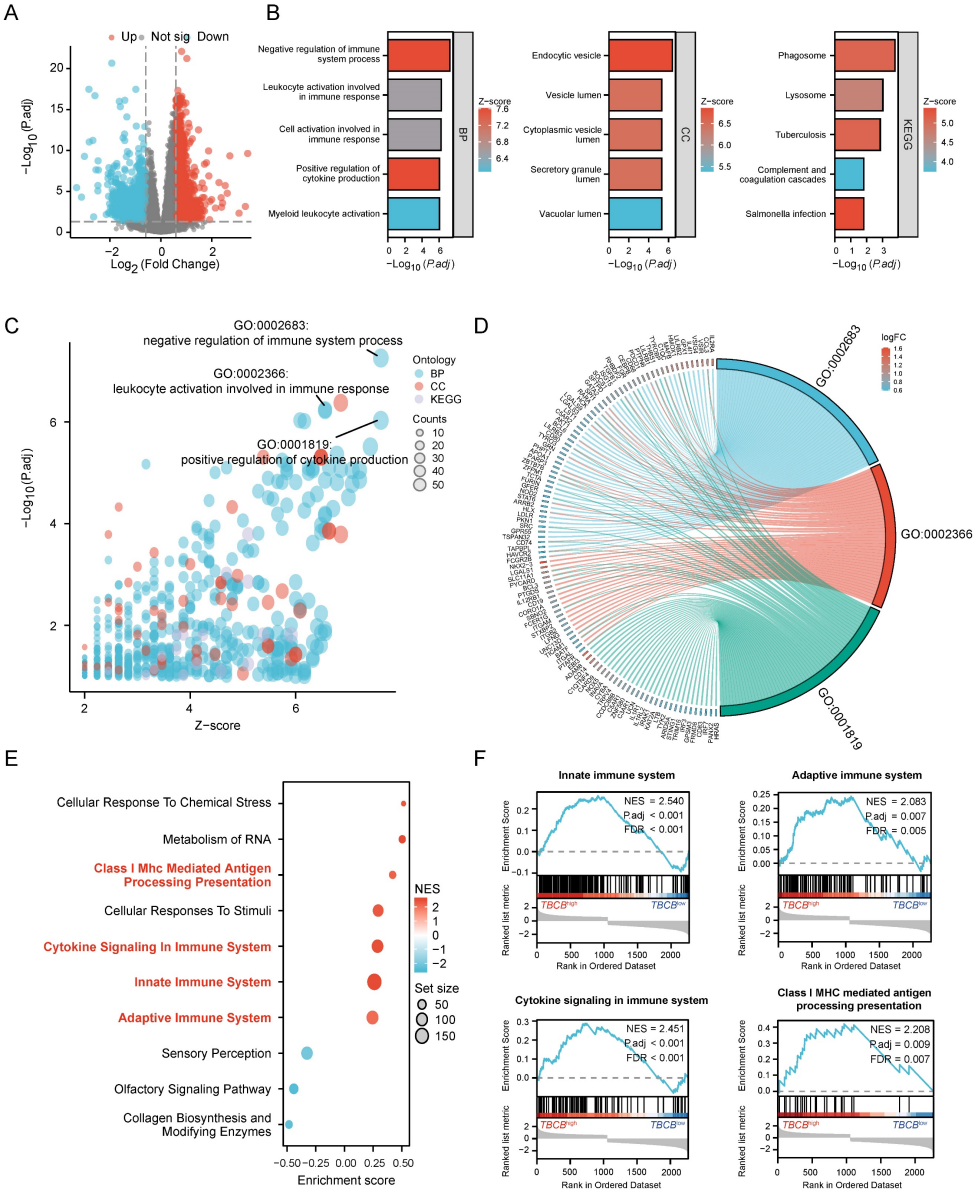
DEGs and their functional pathways enrichment analysis. (A) Volcano plot showing *TBCB*-related DEGs, |log_2_FC| ≥ 0.59, *p*-adjust< 0.05. (B) The bar diagrams display the top five terms for each GO category and KEGG analysis of the up-regulated DEGs, including biological processes (left), cellular components (medium), and KEGG pathways (right). (C-D) Bubble plot (C) and chord plot (D) showing the top 3 BP terms. (E) The first 10 gene sets of GSEA analysis using the Reactome of C2 in MSigDB database. Immune-related gene sets were marked with red. (F) GSEA analysis of immune-related gene sets in up-regulated DEGs.

To investigate the underlying mechanisms of high *TBCB* expression in leukemogenesis, the DEGs analysis was performed in AML populations with high and low *TBCB* expression. A total of 2316 DEGs (|log_2_(FC)| > 0.59, *p*-adjust < 0.05) were identified and displayed in a volcano plot (Figure 3A and Table S3). To thoroughly excavate the intracellular signaling pathway enriched in DEGs, the 1068 up-regulated DEGs were subjected to GO and KEGG analysis. We extracted the top 5 enriched terms from GO and KEGG analysis (*p* < 0.05, Figure 3B and Table S4). Interestingly, we observed that many top terms of BP were related to immune associated pathways (Figure 3C-D and Table S4), including negative regulation of immune system process, leukocyte activation involved in immune response, positive regulation of cytokine production and so forth.

To further analyze immune response pathways involved in DEGs between AML populations with high and low *TBCB* expression, we conducted GSEA enrichment through C2 gene sets in MSigDB database for 2316 DEGs. The top 10 gene sets obtained from Reactome of C2 gene sets were displayed in Figure 3E. GSEA analysis showed enrichment of immune-related signaling pathways (Figure 3F and Table S5) including innate immune system (*p* < 0.001), cytokine signaling in immune system (*p* < 0.001), adaptive immune system (*p* < 0.001) and class I MHC-mediated antigen processing and presentation (*p* < 0.001) in the *high* TBCB expression group. Accordingly, we extrapolated that high *TBCB* expression impinged upon AML may be associated with immunoregulation.

### *TBCB*-related hub genes were involved in immunity

**Figure 4:**
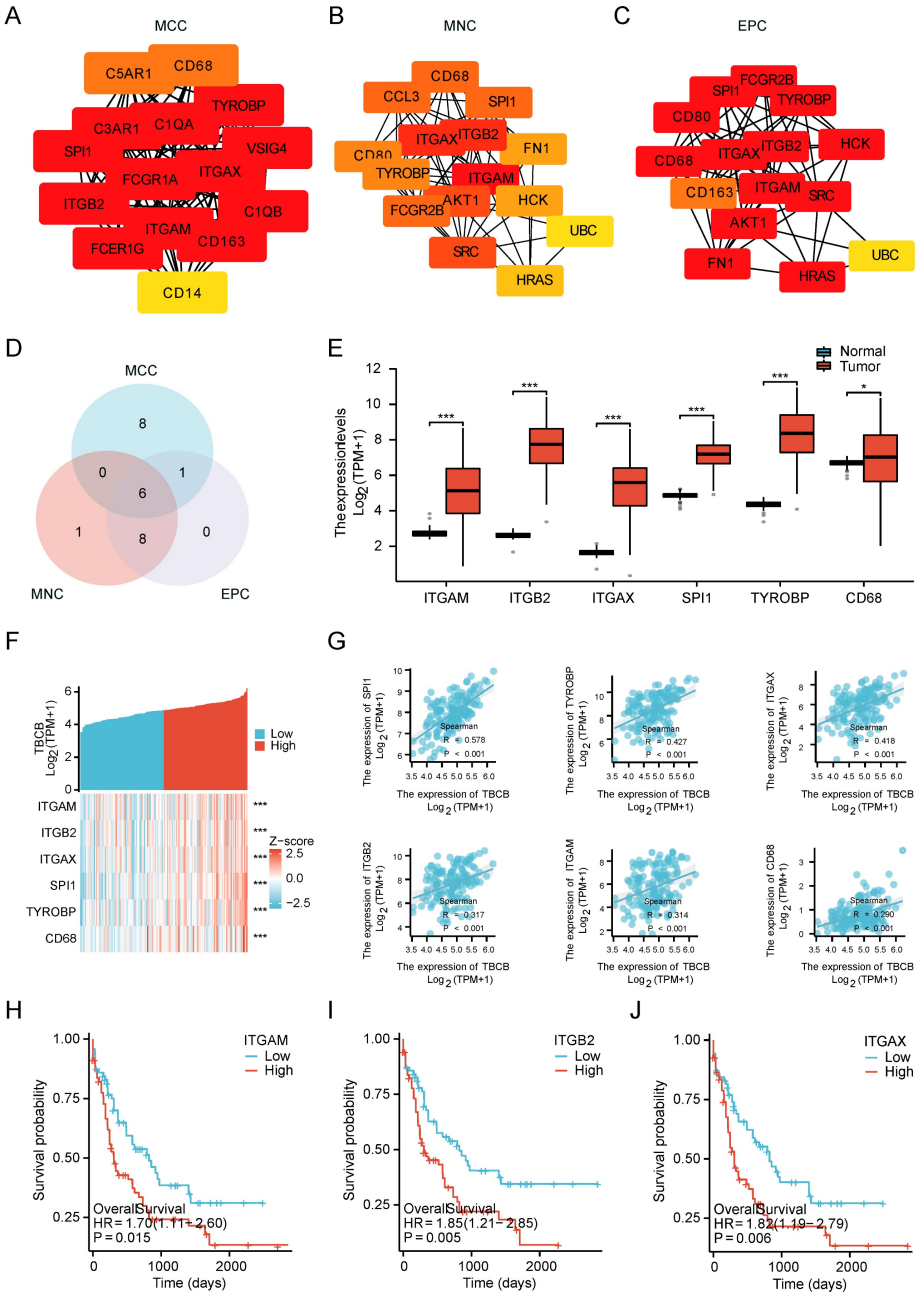
Establishment of PPI network and the clinical significance of hub genes. (A-C) The top15 hub genes were acquired with PPI network on the base of MCC (A), MNC (B) and EPC (C) algorithms. (D) The Venn diagram shows the overlap among the top 15 genes sorted by the three algorithms. (E) Expression levels of six hub genes (ITGAM, ITGB2, ITGAX, SPI1, TYROBP, CD68) in TCGA-LAML and GTEx database. (F-G) Co-expression heat map (F) and correlation scatter plots (G) of TBCB with six hub genes. (H-J) The OS in AML patients, splitting into two populations with high versus low expression in the light of the median expression levels of three hub genes, were created by Kaplan-Meier analysis. ITGAM (H), ITGB2 (I), ITGAX (J). *p < 0.05, ***p < 0.001.

We next used protein-protein interaction (PPI) analyses and co-expression modules to explore which set of proteins may interact with TBCB. Fifteen top hub genes were identified by calculating all of the 1068 up-regulated genes using three different algorithms, namely maximal clique centrality (MCC, Figure 4A), maximum neighborhood component (MNC, Figure 4B), and edge percolated component (EPC, Figure 4C). The above three gene lists shared six common hub genes (ITGAM, ITGB2, ITGAX, SPI1, TYROBP and CD68) (Figure 4D). To validate these hub genes, we determined their expression and prognostic significance. Consistently, all hub genes were highly expressed in AML patients (*p* < 0.05, Figure 4E) and positively correlated with *TBCB* expression (*p* < 0.001, Figure 4F-G). Furthermore, high mRNA levels of three hub genes were also closely linked to poor outcome of AML, which were ITGAM (*p* = 0.015; Figure 4H), ITGB2 (*p* = 0.005; Figure 4I) and ITGAX (*p* = 0.006; Figure 4J). Coincidentally, these three genes are members of the integrin family, which is an integral part of the immune system 45, suggesting that TBCB regulate tumor immune response may be association with integrin pathways.

### Immune-infiltrating NK cell was a presumable factor for the poor prognosis of AML with high* TBCB* expression

**Figure 5:**
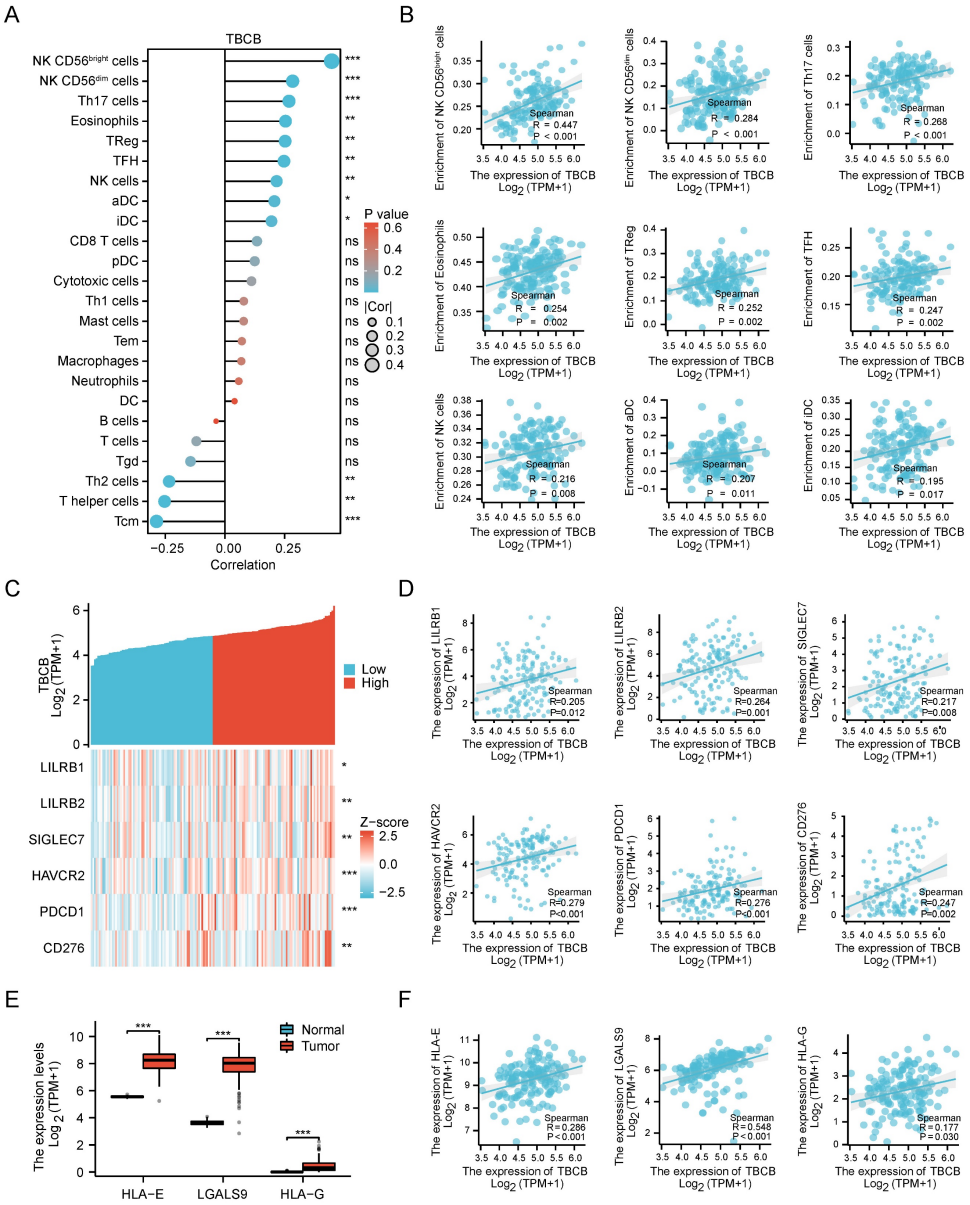
Correlationships between *TBCB* expression level and infiltrating immune cells, immune checkpoint in AML. (A) The relationship between the mRNA level of *TBCB* and twenty four infiltrating immune cells were examined by Spearman's correlation. (B) Correlation scatter plots for nine immune infiltrating cells positively linked to *TBCB* transcription level. (C-D) Co-expression heat map (C) and correlation scatter plots (D) of *TBCB* gene with six momentous immune checkpoint molecules in AML. (E) Expression levels of ligands for inhibitory NK cell receptors (*HLA-E*, *HLA-G* and *LGALS9*) in AML. Data was originated from TCGA-LAML and GTEx database. (F) Correlation scatter plots of *TBCB* gene with three ligands for inhibitory NK cell receptors in AML. *p < 0.05, **p < 0.01, ***p < 0.001.

Since tumor-infiltrating lymphocytes (TILs) have been reported to be prognostic in various tumors 46, 47, we performed correlation analysis between *TBCB* expression and 24 different types of infiltrating immune cell subtypes. Strikingly, the transcriptional level of *TBCB* was positively related to nine immune cells, containing CD56^bright^ NK cells, CD56^dim^ NK cells, Th17 cells, Eosinophils, Treg cells and so forth (*p* < 0.05, Figure 5A). The quantitative analysis for *TBCB* expression level and these immune cells with Spearman's correlation were displayed in Figure 5B. Among these immune infiltrating cells, CD56^bright^ NK cells, which can produce proangiogenic factors and may be induced to decidual-like NK cells in many tumors 48, showed the strongest relevance to *TBCB* expression level. Intriguingly, the enrichment of decidual-like NK cells is inversely linked to the prognosis of patients 48. Moreover, the transcriptional level of *TBCB* was also positively correlated with several immune checkpoint molecules (LILRB1, LILRB2, SIGLEC7, HAVCR2, PDCD1 and CD276; Figure 5C-D), that are NK cell inhibitory receptors. Consistent with this, the transcriptional levels of many ligands for NK cell inhibitory receptors (HLA-E, HLA-G and LGALS9) were significantly increased in AML (Figure 5E) and positively correlated with that of *TBCB* (Figure 5F). These findings indicated that the poor outcome of AML patients with high *TBCB* expression may be related to the immunosuppression on NK cells.

### Transcriptional level of *TBCB* was positively related to those of genes regulating cell proliferation and apoptosis

**Figure 6:**
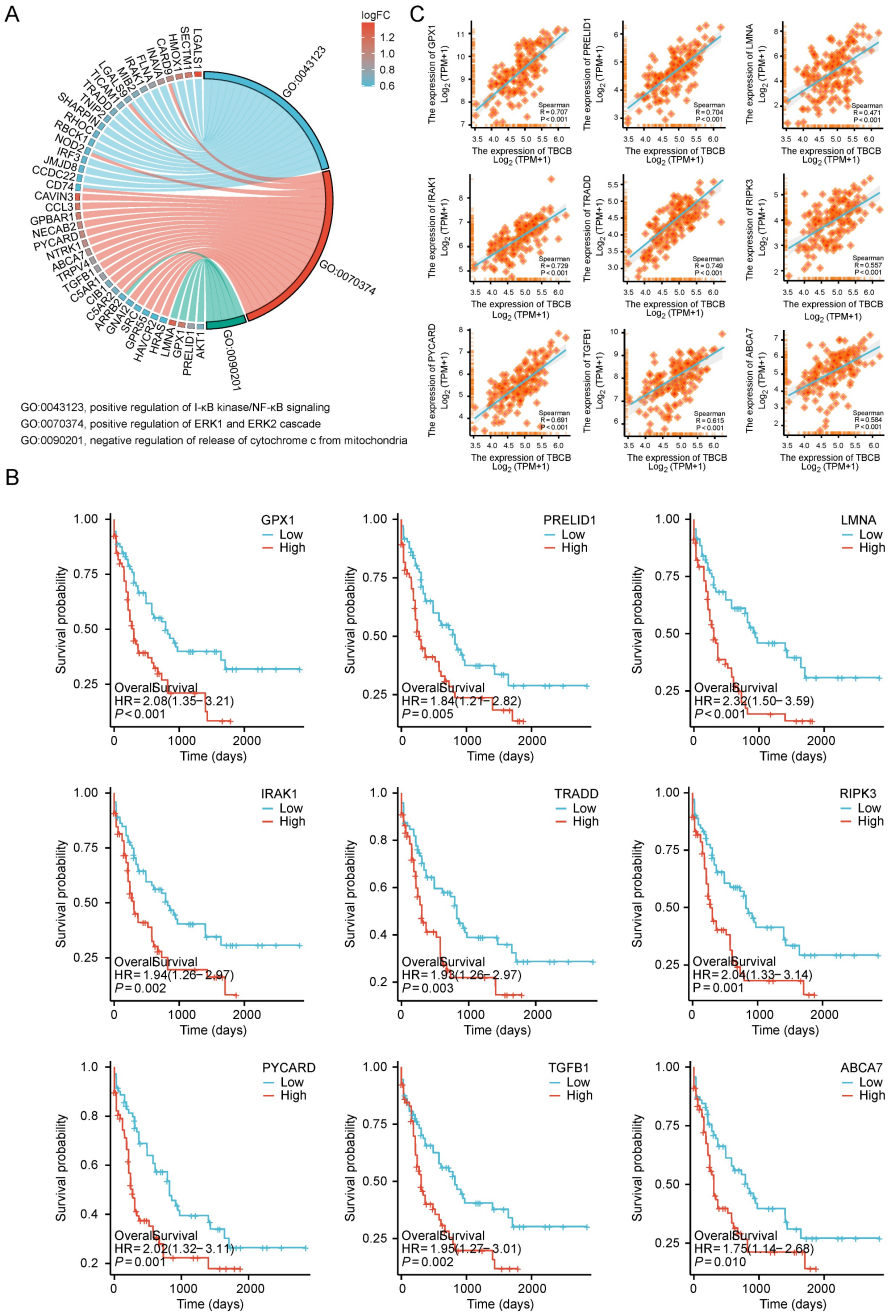
UP-regulated DEGs with high versus low *TBCB* expression involved in cell proliferation and apoptosis gene sets in AML patient. (A) Chord plot showing the relevant GO terms of cell proliferation and apoptosis gene sets. (B-C) The Kaplan-Meier survival curves of OS (B) and correlation analysis with* TBCB* expression (C) for nine genes related to cell proliferation and apoptosis.

Cell proliferation and apoptosis are often used as clinical indicators for tumor prognosis 49. Notably, GO and KEGG analysis showed up-regulated DEGs enriched in signaling pathways involved in positively regulation of NF-κB and ERK1/2 signaling pathways (related to cell proliferation) and negatively regulation of cytochrome C release signaling pathways (related to apoptosis) (*p* < 0.05, Figure 6A and Table S4). In these three signaling pathways, the several molecules (GPX1, RPELID1, LMNA, IRAK1, TRADD, RIPK3, PYCARD, TGFB1, ABCA7), whose high expression levels were markedly associated with the poor prognosis of AML (*p* < 0.01, Figure 6B), had significantly positive correlation with the* TBCB* expression (*p* < 0.001, Figure 6C). Therefore, increased cell proliferation and inhibited apoptosis of tumor cells may also contribute to the poor outcome of AML patients highly expressed *TBCB*.

### Knockdown of TBCB in AML cells suppressed cell proliferation

**Figure 7:**
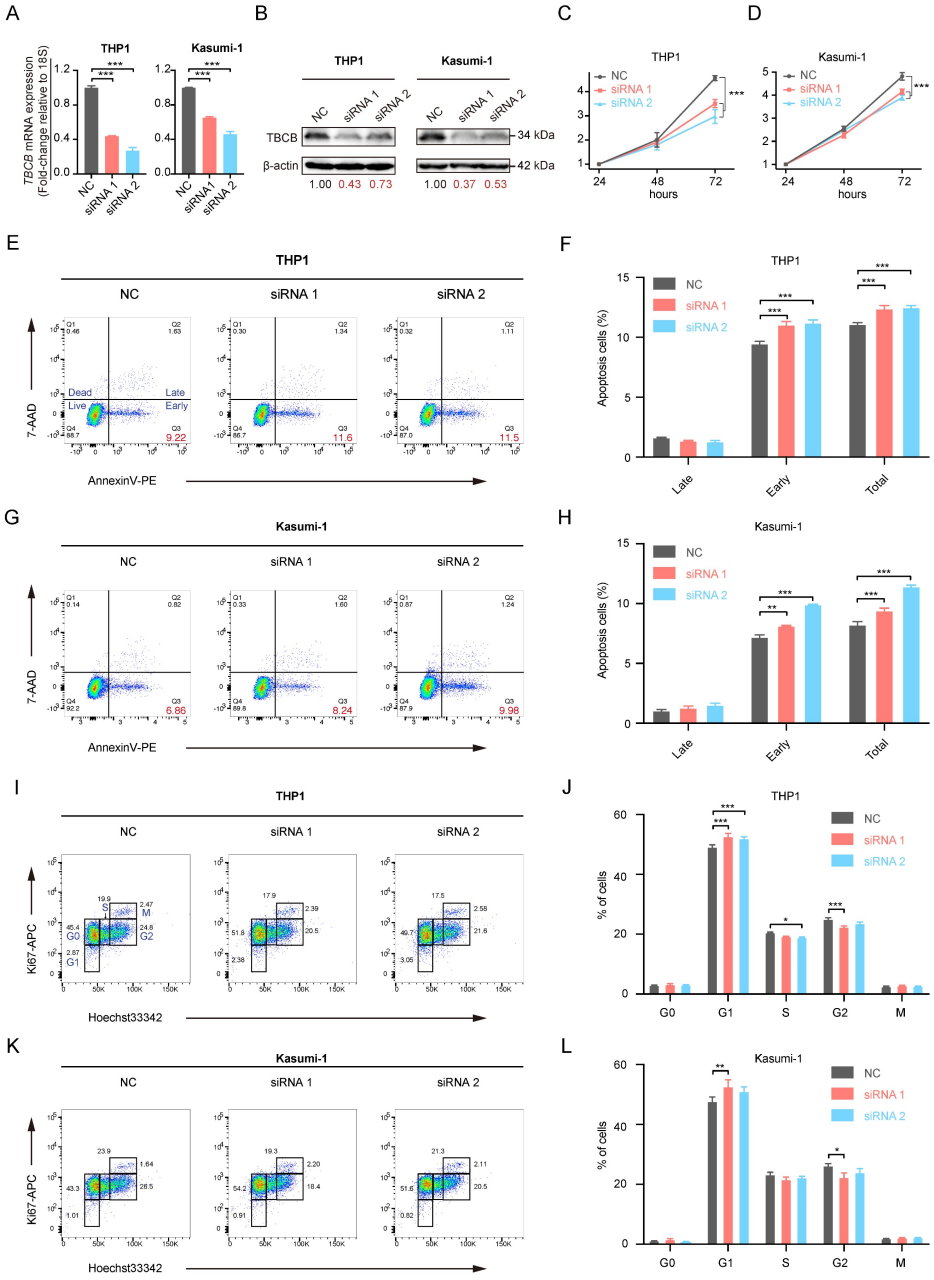
** The effects of TBCB knockdown on cell proliferation in AML human cell lines.** (A) RT-qPCR analysis for transcriptional levels of *TBCB* in AML cell line THP1 (left) and Kasumi-1 (right) transduced with NC-siRNA or siTBCB oligonucleotides. Expression levels are normalized to 18S. (B) Western blotting for TBCB expression in THP1 (left) and Kasumi-1 (right) cell lines transduced with NC-siRNA or siTBCB oligonucleotides. (C-D) Proliferation curves of control and siTBCB groups in THP1 (C) and Kasumi-1 (D) cell lines were measured by CCK8. (E-H) The apoptosis analysis of AML cell lines transfected with siTBCB and NC-siRNA. Representative flow cytometry plots of apoptotic ratio in THP1 (E) and Kasumi-1 (G) cell lines. Statistical analysis of apoptotic ratio in THP1 (F) and Kasumi-1 (H) cell lines. (I-L) The cell cycle analysis of AML cell lines transfected with siTBCB and NC-siRNA. Representative flow cytometric analysis of cell cycle in THP1 (I) and Kasumi-1 (K) cell lines. Quantification of G0, G1, S, G2 and M phases in THP1 (J) and Kasumi-1 (L) cell lines. All statistical values were presented as the means ± SEM. n = 3, *p < 0.05, **p < 0.01, ***p < 0.001, by one-way ANOVA.

To investigate the role of TBCB in AML, we examined the consequences of reducing TBCB expression in AML cell lines by siRNA. The AML cell lines THP1 and Kasumi-1, that highly express TBCB, were transfected with a NC-siRNA and two TBCB siRNAs (siTBCB). RT-qPCR (Figure 7A) and Western blot (Figure 7B) were used to determine the silencing efficiency of the siRNAs. The results suggested that siTBCB effectively decreased the expression of endogenous TBCB in THP1 and Kasumi-1 cells when compared with the control group. In order to examine the effect of TBCB on the cell proliferation of AML cells, we performed CCK-8 assay and found that the silencing TBCB slowed cell proliferation when compared with the NC-siRNA group in both THP1 (Figure 7C) and Kasumi-1 (Figure 7D) cells. Apoptosis and cell cycle analysis were conducted to investigate the mechanisms by which the decreased TBCB expression inhibited cell proliferation. The rates of early and total apoptotic cells were increased in siTBCB groups, and were significantly higher than those of the control group (Figure 7E-H). Furthermore, we also observed that the cell cycles were arrested when TBCB was suppressed. The percentages of G1 phase cells were increased significantly in siTBCB groups when compared with that of the control group, while the percentages of S and G2 phase cells were slightly decreased (Figure 7I-L). These findings suggested that the down-regulation of TBCB suppressed cell proliferation by enhancing the apoptosis rate and arresting cell cycle, further supporting our results obtained from database analyses that high expression of *TBCB* increased tumor cell proliferation and inhibited apoptosis of tumor cells in AML patients.

### AML cells highly expressed *TBCB* were sensitive to midostaurin but resistant to cytarabine

**Figure 8:**
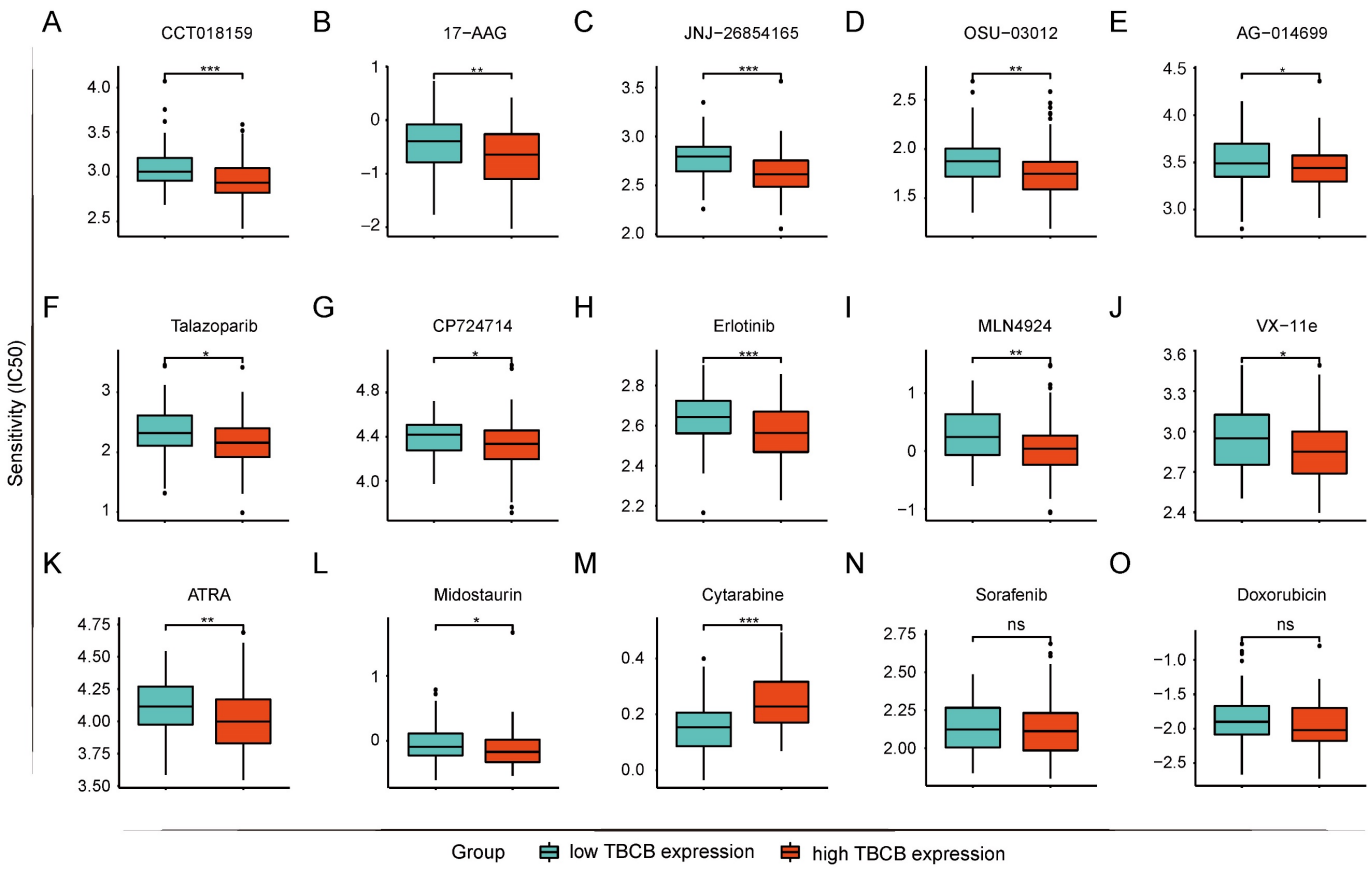
Prediction of drug sensitivity based on *TBCB* expression. (A) CCT018159. (B) 17-AAG. (C) JNJ-268541653. (D) OSU-03012. (E) AG-0140699. (F) Talazoparib. (G) CP724714. (H) Erlotinib. (I) MLN4924. (J) VX-11e. (K) ATRA. (L) Midostaurin. (M) Cytarabine. (N) Sorafenib. (O) Doxorubicin. *p < 0.05, *p < 0.01, ***p < 0.001.

The prediction of drug susceptibility was executed for the two AML populations with high and low expression of *TBCB*. Collectively, group with high *TBCB* expression was shown to sensitive to HSP90 inhibitors (CCT018159 and 17-AAG; Figure 8A-B), p53 activator (JNJ-26854165; Figure 8C), pyruvate Dehydrogenase Kinase 1 (PDK1) inhibitor (OSU-03012; Figure 8D), poly (ADP-ribose) polymerase (PARP) inhibitors (AG-0140699 and Talazoparib; Figure 8E-F), epidermal growth factor receptor (EGFR) inhibitors (CP724714 and Erlotinib; Figure 8G-H), NEDD8 inhibitor (MLN4924; Figure 8I), ERK2 inhibitor (VX-11e; Figure 8J), and so on. In contrast, these cells were resistant to mTOR inhibitors (Rapamycin and AZD8055; Figure S2A-B), tyrosine kinase inhibitors (Imatinib and Dasatinib; Figure S2C-D), PI3K inhibitors (GSK2126458, PI-103, PIK-93 and ZSTK474; Figure S2E-H). Several drugs used clinically for AML-directed therapies, such as ATRA (Figure 8K) and Midostaurin (Figure 8L), exhibited a lower IC50 in high *TBCB* expression group, while Cytarabine (Figure 8M) had a higher IC50. In addition, there was no strong discrepancy in response to Sorafenib (Figure 8N) and Doxorubicin (Figure 8O) between the two AML populations with high versus low *TBCB* expression. The drug susceptibility analysis results may be helpful for drug selection of AML patients with differential TBCB expression.

## Discussion

Acute myeloid leukemia (AML), with uncontrolled overproduction of myeloid cells, is one of the most threatening hematological malignancies 50. Rapid progresses of disease and strong resistance to chemotherapy engender high incidence of relapse rate and extremely adverse prognosis in AML patients 51. Although many new targeted drugs have been approved for the clinical treatment of AML, with improved remission rate and prognosis of patients since 2017 52, the high heterogeneity of the disease leads to a pressing necessity for more comprehensive and effective prognostic and therapeutic targets of AML.

Microtubules (MTs) are cytoskeletal polymers pivotal for eukaryotic activities, including but not limited to cell division, migration and intracellular trafficking 53, 54. The excessive proliferation of tumor cells depends on the rapid polymerization and depolymerization of tubulin 55. As a conserved tubulin folding cofactor, TBCB regulates the assembly and disassembly of α-β tubulin heterodimers 56, which forms a physiological dimer with TBCE and plays an important role in microtubule biosynthesis 57. When *TBCB* is overexpressed, it will lead to microtubule depolymerization and mitosis disorder, thus resulting in disease 58. However, the role of TBCB in the treatment and prognosis of AML is still not defined.

In this study, we identified *TBCB* as a potential prognostic marker in AML. High mRNA level of *TBCB* was obviously related to unfavorable prognosis, as demonstrated by both univariate and multivariate regression analysis. As predicted, high mRNA level of *TBCB* was consistent with elevated WBC count, increased proportion of PB and BM blasts, leading us to speculate that the abnormal high level of *TBCB* acts as a positive regulatory factor of AML for promoting the cell proliferation. The up-regulated DEGs were enriched in signaling pathways relevant for cell proliferation activation and apoptosis inhibition via GO and KEGG analysis. We decreased the expression level of TBCB in AML cell lines by siRNAs and found that the cells obtained slower growth, increased apoptosis and cell cycle arrest. To sum up, it was conceivable that the poor outcome of AML patients bearing high* TBCB* expression may be related to cell proliferation activation and apoptosis inhibition, but the specific underlying mechanism demands an in-depth study.

Moreover, transcriptional level of *TBCB* was positively linked to those of immune-related molecules, as revealed by PPI network analysis, GO and GSEA enrichment analysis. The high expression of three hub genes (LILRB2, SIGLEC7, and PDCD1) which are significantly associated with TBCB was also obviously linked to poor prognosis in AML. Moreover, the *TBCB* expression was associated with immune infiltration cells in AML, particularly by CD56^bright^ NK cells with low cytotoxicity 59. Interestingly, CD56^bright^ CD16^-^ NK cells are the principal components of NK cells in various immune tolerance organs 60 and their function were found to hampered in tumor immune environments 48. Therefore, we reasonably speculated that the tumor microenvironment infiltrated with a large number of CD56^bright^ NK of AML patients owned high expression of *TBCB* might cause immune escape of tumor cells, ultimately leading to an unfavorable outcome. Taking a step further, we found that the transcription levels of NK cell inhibitory receptors and their ligands were positively related to that of* TBCB*, and their high expression also predicted poor prognosis. These results suggest that *TBCB* may be involved in regulating the function of NK cells and modulating the immune microenvironment of AML. Targeted drugs for NK inhibitory receptors have garnered a great deal of attention 61, 62, and these drugs could potentially serve as a treatment alternative for AML patients with high *TBCB* expression by alleviating the immunosuppression of NK cells.

We also examined the drug sensitivity of clinical drugs in AML cells with high or low transcriptional levels of *TBCB*. Our findings exhibited that AML cells highly expressed *TBCB* were sensitive to ATRA and midostaurin, but resistant to cytarabine, dasatinib and imatinib. Therefore, such knowledge for clinical drug usages should be considered when making a choice in the combined use of AML drugs.

## Conclusion

In this study, we provided evidence that *TBCB* is a potential prognostic marker and therapeutic target for AMLs. Our findings suggested that TBCB might be involved in regulating AML cell proliferation, the immune microenvironment of AML, and the function of NK cells. The underlying mechanism of TBCB in regulating tumor cell proliferation and NK cell-mediated immune escape in AML needs further in-depth studies.

## Supplementary Material

Supplementary figures.

Supplementary tables.

## Figures and Tables

**Table 1:** Clinical characteristics with diverse *TBCB* expression levels in AML patients

Characteristics	Low expression of *TBCB*	High expression of *TBCB*	P value
n	75	75	
Gender, n (%)	75	75	0.412
Female	31 (20.7%)	36 (24%)	
Male	44 (29.3%)	39 (26%)	
Age, n (%)	75	75	0.069
<= 60	49 (32.7%)	38 (25.3%)	
> 60	26 (17.3%)	37 (24.7%)	
WBC count(x10^9/L), n (%) ^i^	74	75	0.040
<= 20	44 (29.5%)	32 (21.5%)	
> 20	30 (20.1%)	43 (28.9%)	
BM blasts(%), n (%)	75	75	0.012
<= 20	37 (24.7%)	22 (14.7%)	
> 20	38 (25.3%)	53 (35.3%)	
PB blasts(%), n (%)	75	75	0.141
<= 70	40 (26.7%)	31 (20.7%)	
> 70	35 (23.3%)	44 (29.3%)	
Cytogenetic risk, n (%) ^ii^	75	73	0.152
Favorable	19 (12.8%)	11 (7.4%)	
Intermediate/normal	36 (24.3%)	46 (31.1%)	
Poor	20 (13.5%)	16 (10.8%)	
FAB classifications, n (%) ^iii^	75	74	0.004
M0	12 (8.1%)	3 (2%)	
M1&M2	29 (19.5%)	44 (29.5%)	
M3	9 (6%)	5 (3.4%)	
M4	18 (12.1%)	11 (7.4%)	
M5	4 (2.7%)	11 (7.4%)	
M7&M6	3 (2%)	0 (0%)	
Cytogenetics, n (%) ^iv^	68	66	0.170
Normal	29 (21.6%)	40 (29.9%)	
+8	7 (5.2%)	1 (0.7%)	
del(5)&del(7)	3 (2.2%)	4 (3%)	
inv(16)	5 (3.7%)	3 (2.2%)	
t(8;21)&t(9;11)&t(15;17)	10 (7.5%)	8 (6%)	
Complex	14 (10.4%)	10 (7.5%)	
FLT3 mutation, n (%) ^v^	73	73	0.020
Negative	57 (39%)	44 (30.1%)	
Positive	16 (11%)	29 (19.9%)	
IDH1 R132 mutation, n (%) ^vi^	74	74	0.772
Negative	67 (45.3%)	68 (45.9%)	
Positive	7 (4.7%)	6 (4.1%)	
IDH1 R140 mutation, n (%) ^vii^	74	74	1.000
Negative	68 (45.9%)	68 (45.9%)	
Positive	6 (4.1%)	6 (4.1%)	
IDH1 R172 mutation, n (%) ^viii^	74	74	0.477
Negative	72 (48.6%)	74 (50%)	
Positive	2 (1.4%)	0 (0%)	
RAS mutation, n (%) ^ix^	74	75	0.702
Negative	69 (46.3%)	72 (48.3%)	
Positive	5 (3.4%)	3 (2%)	
NPM1 mutation, n (%) ^x^	74	75	0.083
Negative	62 (41.6%)	54 (36.2%)	
Positive	12 (8.1%)	21 (14.1%)	

^i^ WBC count was missing in patient with sample ID TCGA-AB-2991 (abbreviated as 2991) ^ii^ Information on cytogenetic risk were missing in patients with sample ID 2810 and 2895 ^iii^ Information on FAB classifications were missing in patient with sample ID 2941 ^iv^ Clinical information on cytogenetic risk were missing in patients with sample ID 2810, 2828, 2834, 2842, 2843, 2844, 2849, 2865, 2877, 2888, 2893, 2895, 2916, 2931, 2944 and 2946 ^v^ FLT3 mutation was not detected in patients with sample ID 2869, 2995, 2996 and 3002 ^vi^ IDH R132 mutation was not detected in patients with sample ID 2932 and 2991 ^vii^ IDH R140 mutation was not detected in patients with sample ID 2869 and 2995 ^viii^ IDH R172 mutation was not detected in patients with sample ID 2869 and 2995 ^ix^ RAS mutation was not detected in patient with sample ID 2995 ^x^ NPM1 mutation was not detected in patient with sample ID 2995

## Abbreviations

AML: acute myeloid leukemia
AUC: area under curve
CI: confidence intervals
DEG: differential expression genes
EGFR: epidermal growth factor receptor
EPC: edge percolated component
ERK: extracellular-signal regulated kinases
GO: gene ontology
GSEA: gene set enrichment analysis
HR: hazard ratios
HSCT: hematopoietic stem cell transplantation
KEGG: kyoto encyclopedia of genes and genomes
MCC: maximal clique centrality
MM: multiple myeloma
MNC: maximum neighborhood component
MTAs: microtubule targeted agents
NEDD8: neural precursor cells-expressed developmentally down-regulated protein-8
OS: overall survival
PARP: poly (ADP-ribose) polymerase
PDK1: pyruvate dehydrogenase kinase 1
PI3K: phosphoinositide 3-kinase
PPI: protein-protein interaction
ROC: receiver operating characteristic
RT-qPCR: real-time quantitative polymerase chain reaction
TCGA: The Cancer Genome Atlas
TPM: transcript per million
UCSC: University of California Santa Cruz
