# Supplementary material for: High Expression of Microtubule-associated Protein TBCB Predicts Adverse Outcome and Immunosuppression in Acute Myeloid Leukemia
Source: J Cancer. 2023 Jun 12;14(10):1707–24. doi: 10.7150/jca.84215 (PMC10355208; doi:10.7150/jca.84215)
Supplement: Supplementary file 1 — Supplementary figures. [file jcav14p1707s1.pdf]

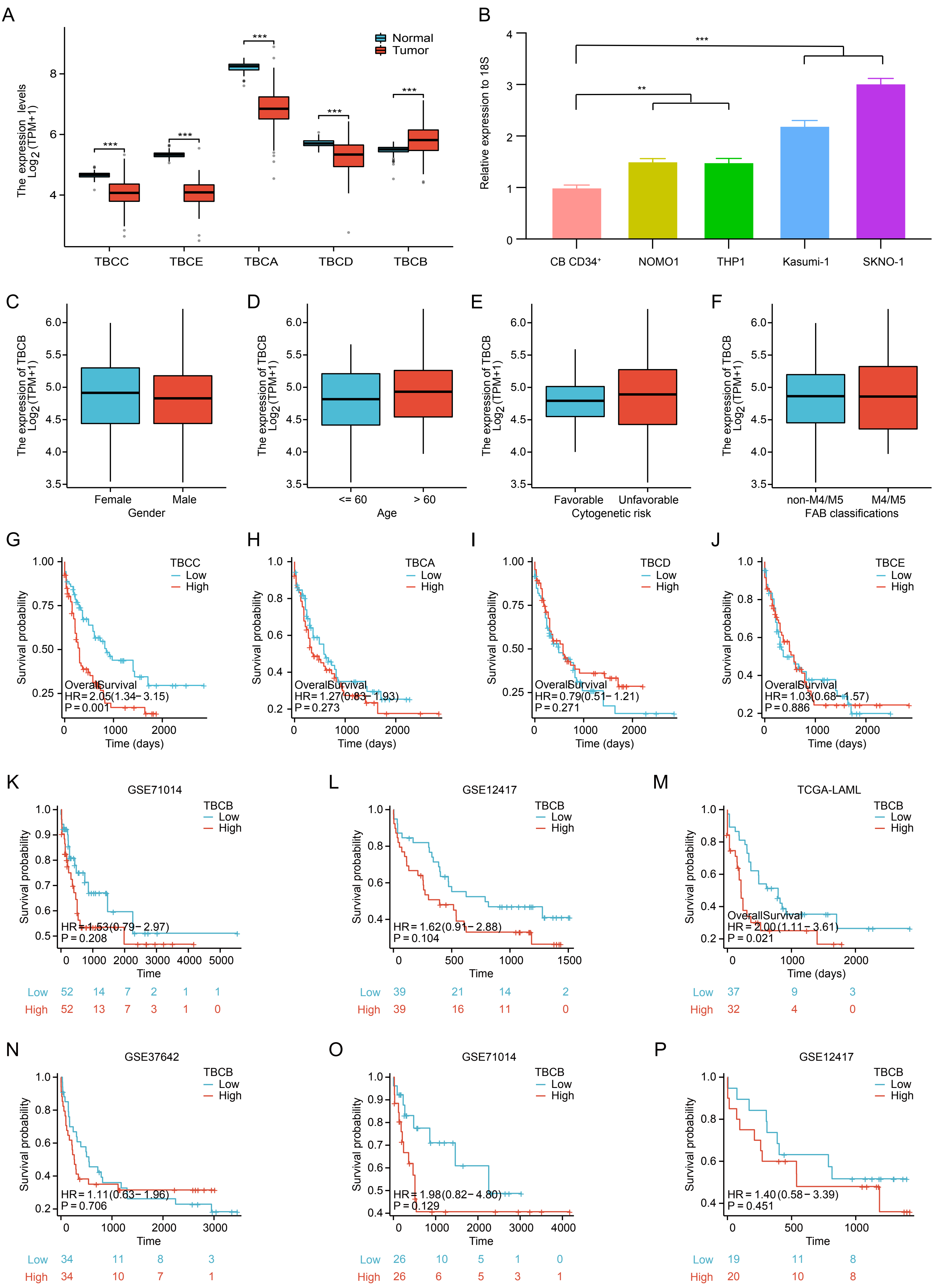
A
The expression levels
Log2 (TPM+1)
Normal
Tumor
***
TBCC
TBCE
TBCA
TBCD
TBCB
B
Relative expression to 18S
***
**
CB CD34+
NOMO1
THP1
Kasumi-1
SKNO-1
C
The expression of TBCB
Log2 (TPM+1)
Female
Male
Gender
D
<= 60
> 60
Age
E
Favorable
Unfavorable
Cytogenetic risk
F
non-M4/M5
M4/M5
FAB classifications
G
Survival probability
TBCC
Low
High
OverallSurvival
HR=2.05(1.34−3.15)
P=0.001
Time (days)
H
TBCA
Low
High
OverallSurvival
HR=1.27(0.83−1.93)
P=0.273
Time (days)
I
TBCD
Low
High
OverallSurvival
HR=0.79(0.51−1.21)
P=0.271
Time (days)
J
TBCE
Low
High
OverallSurvival
HR=1.03(0.68−1.57)
P=0.886
Time (days)
K
GSE71014
TBCB
Low
High
HR=1.53(0.79−2.97)
P=0.208
Time
Low 52 14 7 2 1 1
High 52 13 7 3 1 0
L
GSE12417
TBCB
Low
High
HR=1.62(0.91−2.88)
P=0.104
Time
Low 39 21 14 2
High 39 16 11 0
M
TCGA-LAML
TBCB
Low
High
OverallSurvival
HR=2.00(1.11−3.61)
P=0.021
Time (days)
Low 37 9 3
High 32 4 0
N
GSE37642
TBCB
Low
High
HR=1.11(0.63−1.96)
P=0.706
Time
Low 34 11 8 3
High 34 10 7 1
O
GSE71014
TBCB
Low
High
HR=1.98(0.82−4.80)
P=0.129
Time
Low 26 10 5 1 0
High 26 6 5 3 1
P
GSE12417
TBCB
Low
High
HR=1.40(0.58−3.39)
P=0.451
Time
Low 19 11 8
High 20 10 8

Figure S1

Transcriptional level of TBCs and their prognostic analysis in AML patients.

(A) The transcriptional levels of TBCs in AML patients and normal subjects. Data were originated from TCGA-LAML and GTEx.

(B) The mRNA levels of *TBCB* in CB CD34$^+$ cells and AML cell lines by RT-qPCR. Expression levels are normalized to 18S. CB, cord blood.

(C-F) No association between the mRNA levels of *TBCB* and the gender, age, cytogenetic risk and FAB classifications in AML patients.

(G-J) Kaplan–Meier survival curves of OS were drawn from AML patients grouped into high and low populations as per the median expression of *TBCC* (G), *TBCA* (H), *TBCD* (I) and *TBCE* (J). Data was originated from TCGA-LAML dataset.

(K-L) Kaplan–Meier survival curve of OS was delineated for AML patients grouped into high versus low expressed populations in line accordance with the median expression of *TBCB*. Data was originated from GSE71014 (K) and GSE12417 (L).

(M-P) Kaplan–Meier survival curve of OS was delineated for AML patients grouped by quartile boundaries of *TBCB* expression levels. Data was originated from TCGA-LAML (M), GSE37642 (N) GSE71014 (O) and GSE12417 (P).

One-way ANOVA was conducted with the results of RT-qPCR. *$p < 0.05$, **$p < 0.01$, ***$p < 0.001$.

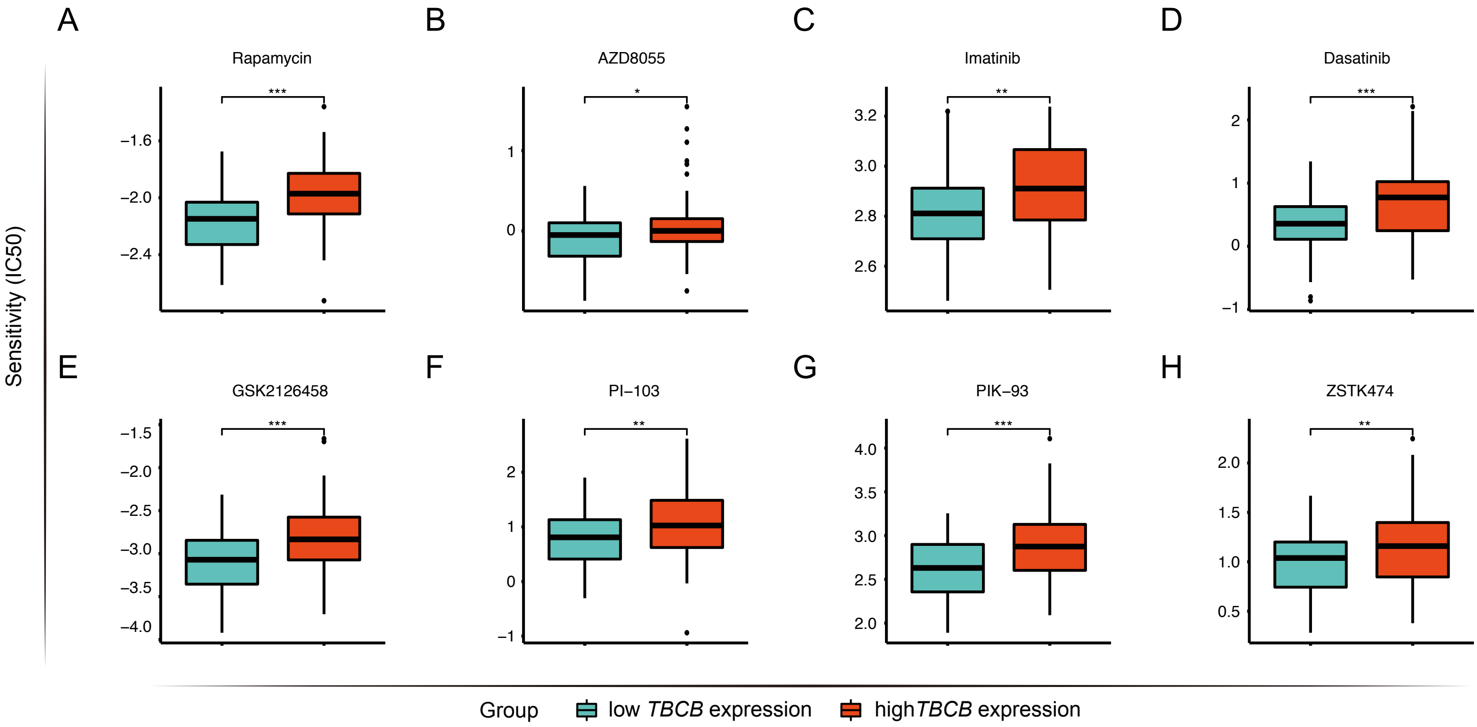

Figure S2

Prediction of drug resistance in high and low *TBCB* expression populations.

(A) Rapamycin. (B) AZD8055. (C) Imatinib. (D) Dasatinib. (E) GSK2126458. (F) PI-103. (G) PIK-93. (H) ZSTK474. *p < 0.05, **p < 0.01, ***p < 0.001.
